# Google search data as a novel adjunct to patient and public involvement in rheumatology research

**DOI:** 10.1007/s00296-020-04723-0

**Published:** 2020-10-19

**Authors:** Mrinalini Dey, Sizheng Steven Zhao

**Affiliations:** 1 Institute of Life Course and Medical Sciences, University of Liverpool, Brownlow Hill, Liverpool, L69 3BX UK; 2 Department of Rheumatology, Aintree Hospital, Liverpool University Hospitals NHS Foundation Trust, Lower Lane, Liverpool, L9 7AL UK

**Keywords:** Patient involvement, Rheumatology, Arthritis, Rheumatoid arthritis, Data visualisation, Google

## Abstract

Patient and public involvement is essential in the design and implementation of research studies to ensure research remains relevant and in line with public priorities. Public views on a given area of research may be sought via platforms such as focus groups or surveys. Here, we present the use of an openly available Google search data query tool, which may be used alongside traditional forms of patient and public involvement in research to highlight public perceptions and priorities. We used an online search query tool (“AnswerThePublic.com”) to explore public Google searches relating to “arthritis,” and an exemplar rheumatic disease, “rheumatoid arthritis.” The most common searches relating to these diseases included quality of life, treatment, prognosis, as well as impacts on life, including work. However, they also reveal concerns that may be more difficult to elicit in face-to-face focus groups, such as questions on alcohol consumption in arthritis, and impacts on mental health. Using public search engine data in research, alongside the important traditional methods of patient and public involvement, is a cost-effective and time-efficient method of gauging public views and concerns on a given topic. It may facilitate broad scoping searches of public priorities and help to guide future research questions.

## Introduction

Patient and public involvement (PPI) is integral to the design and prioritisation of research studies. The National Institute for Health Research (NIHR) defines public involvement in research as “research being carried out ‘with’ or ‘by’ members of the public rather than ‘to’, ‘about’ or ‘for’ them” [1]. PPI are typically conducted by funders and researchers to prioritise research questions, and offering advice as part of a project steering group. The term ‘public’ denotes a wide range of individuals, including patients, potential patients, carers, people using health and social care services, and those who represent them [1]. It is important to distinguish between the views of the public, and those with a professional role in health and social care, especially when considering research design and prioritisation. This facilitates optimal impact and improved enrolment to studies, especially if those with the condition under study are involved from the study’s inception [2]. In the rheumatic diseases, undertaking PPI ensures research remains patient-centred and clinically relevant, across a range of chronic and often debilitating conditions, treated with a wide range of drugs, including immunosuppressants and analgesics.

There is ample evidence demonstrating that PPI is necessary to optimise research design and output [2–4]. However, at the earliest stages of designing a study, it can be useful to gain a broad overview of public perceptions of a given topic, ensuring increased relevance and potential for wider benefit. Furthermore, it can be difficult to have access to focus groups, especially for junior researchers and clinicians. This has been made all the more difficult by social-distancing measures implemented worldwide due to the COVID-19 pandemic, which are likely to have lasting consequences for months to years. In outpatient specialties such as rheumatology, a quick and easy method of gauging public priorities for research can be through questionnaires and surveys conducted in clinic, but access to this may also be limited in the short-medium term due to increased use of telephone consultations.

There is no replacement for PPI, for example in the form of stakeholder involvement, individual interviews, and focus groups, which will always be necessary to ensure adequate prioritisation and relevance of research. However, large-scale search query tools can overcome some of the issues described above. These openly available online tools are able to collate and display keyword suggestions and predictions based on searches within Google, which is used for 78% of internet searches. With 3.5 billion searches conducted per day through Google, summarising searches this way can provide an excellent overview of public concerns and queries on a given topic [5]. They are already commonplace in marketing, to ensure content is relevant and up-to-date. An example of such a tool includes “AnswerThePublic.com” [6], which is able to fetch data on phrases and questions entered into Google, featuring certain keywords (as entered into the tool by the user), Results are presented in both a tabulated and infographic format, making it efficient to ascertain search patterns and popular phrases.

In healthcare, search query tools may not only serve to confirm prior knowledge from PPI and clinician perceptions, but also reveals searches on more sensitive topics, such as opioid dependence and psychological impact of rheumatic disease, which service-users may not necessarily wish to discuss face-to-face.

We aimed to demonstrate the utility of a search query tool to complement PPI in the rheumatic diseases, using an exemplar rheumatic disease, rheumatoid arthritis.

## Methods

We used an openly available search query tool to collate and map Google searches for two separate terms: “arthritis” and “rheumatoid arthritis.” We opted to use “AnswerThePublic.com”, due to ease of use and the resulting visual format of the results. Data on searches performed in Google are presented in real-time, so results are up-to-date at the time of using the search query tool. Since the results at any given time are identical regardless of the individual conducting the search, one author conducted the search for the chosen terms. Search queries are based on Google searches in the region in which it is accessed (e.g. UK); therefore, factors such as the user’s own Google search history are irrelevant.

We first explored public Google searches for “arthritis” to gauge a broad overview of public queries on one of the most commonly-used terms in rheumatology, also used by patients and public. We then assessed search suggestions for “rheumatoid arthritis,” as an exemplar rheumatic disease. Rheumatoid arthritis was selected as it is the most prevalent chronic inflammatory arthritis, and, therefore, likely to be a commonly-searched term in this field [7]. All searches were performed on 19 May 2020.

The output of results for each of the above two queries were delivered in the following categories: questions on the term; prepositions (i.e. searches incorporating additional terms such as “with” and “near”); comparisons (i.e. searches incorporating additional terms such as “and” and “versus”). Results were presented in lists as well as data visualisation images. Search query data for the UK, English language only, were interrogated.

## Results

**Fig. 1:**
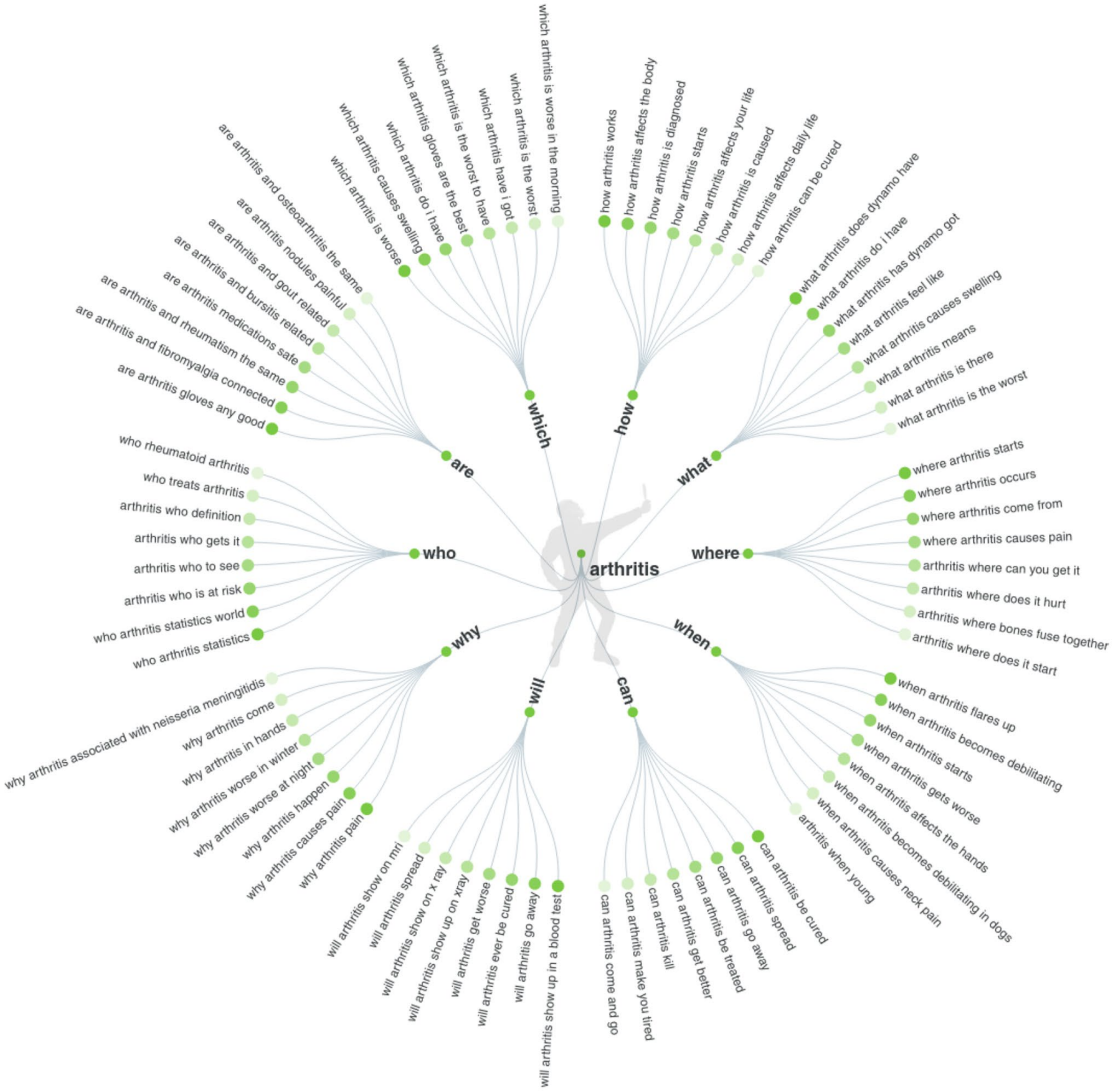
Data visualisation of the 8 most common questions asked on “arthritis,” using the interrogative words: are, which, how, what, where, when, can, will, why, who. Darker green denotes an increased number of searches, while paler green denotes fewer searches

Figure 1 displays the results for search terms relating to questions on “arthritis,” with up to eight of the most common terms shown for each, as an example of output produced by the search query tool. Within each sub-category, the darker stems denote more popular search terms, while paler stems denote less popular search terms.

Subsequent searches are displayed in Tables 1, 2 and 3. Table 1 displays results for search terms relating to prepositions and comparisons to “arthritis”.

**Table 1:** Top: the eight most common search terms on “arthritis,” using the prepositions: can, without, is with, for, to, near, bottom: the 8 most common search terms on “arthritis,” using the comparators: vs, and, like, or, versus. Searches are ordered in popularity vertically down each column, with the most popular at the top

Searches on “arthritis”: prepositions						
With	Near	Without	Can	To	Is	For
Arthritis with rash	Arthritis near me	Arthritis without swelling	Arthritis can opener	Arthritis to the knee	Arthritis is an autoimmune disease	Arthritis for dogs
Arthritis with ulcerative colitis	Arthritis near ear	Arthritis without pain	Arthritis can opener UK	Arthritis to the spine	Arthritis is wrist	Arthritis for cats
Arthritis without swelling	Arthritis near heart	Arthritis without medication	Arthritis can be cured	Arthritis to eat	Arthritis is it hereditary	Arthritis for dummies
Arthritis with psoriasis	Arthritis near thumb	Arthritis without joint pain	Arthritis can you die	Arthritis to fingers	Arthritis is hands	Arthritis for young adults
Arthritis without pain	Arthritis near collar bone	Arthritis without stiffness	Arthritis can I claim pip	Arthritis to hands	Arthritis is curable	Arthritis for dogs medication
Arthritis with skin rash	Arthritis in elbow	Arthritis without fever	Arthritis can you get disability	Arthritis to avoid food	Arthritis is thumb joint	Arthritis for dogs treatment
Arthritis with Crohn's	Arthritis near groin	Arthritis without treatment	Arthritis can I claim disability allowance	Arthritis to lower back	Arthritis is killing me	Arthritis for dogs home remedies
Arthritis with Crohn's disease	Arthritis in spine	Arthritis without swelling or redness	Arthritis can cause fever	Arthritis to avoid	Arthritis is disability	Arthritis for cats natural remedies

Searches on “arthritis”: comparators						
	Arthritis vs gout	Arthritis and tomatoes	Arthritis like conditions	Arthritis or joint pain	Arthritis versus arthritis	
	Arthritis vs arthralgia	Arthritis and alcohol	Arthritis like pain that moves around	Arthritis or gout	Arthritis versus rheumatoid arthritis	
	Arthritis vs arthrosis	Arthritis and diet	Arthritis like symptoms but not arthritis	Arthritis or RSI	Arthritis versus osteoarthritis	
	Arthritis vs MS	Arthritis and COVID	Arthritis like diseases	Arthritis or bone cancer	Arthritis versus fibromyalgia	
	Arthritis vs rheumatism	Arthritis and fatigue	Arthritis like pain	Arthritis or osteoarthritis	Arthritis versus gout	
	Arthritis vs tendonitis	Arthritis and exercise	Arthritis like pain in hands	Arthritis or fibromyalgia	Arthritis versus bursitis	
	Arthritis vs bursitis	Arthritis and rheumatology	Arthritis like pain postpartum	Arthritis or bursitis	Arthritis versus arthrosis	
	Arthritis vs osteoarthritis	Arthritis and turmeric	Arthritis like pain in hands during pregnancy	Arthritis or cancer	Arthritis versus tendonitis

**Table 2:** The most common questions asked on “rheumatoid arthritis,” using the interrogative words: who, how, when, are, will, why, what, can, where, which. Searches are ordered in popularity vertically down each column, with the most popular at the top

Searches on “rheumatoid arthritis”: questions									
How	When	Are	Will	Why	What	Can	Where	Who	Which
How rheumatoid arthritis affects daily life	When rheumatoid arthritis turns deadly	Are rheumatoid arthritis patients immunosuppressed	Will rheumatoid arthritis show up on x-ray	Why rheumatoid arthritis occurs	What’s rheumatoid arthritis	Can rheumatoid arthritis affect your jaw	Where does rheumatoid arthritis start	Who rheumatoid arthritis	which joints rheumatoid arthritis
How rheumatoid arthritis affects the body	When rheumatoid arthritis is active	Are rheumatoid arthritis and psoriasis related	Will rheumatoid arthritis show up in a blood test	Why rheumatoid arthritis is an autoimmune disease	What rheumatoid arthritis feels like	Can rheumatoid arthritis affect your eyes	Where does rheumatoid arthritis affect	Who gets rheumatoid arthritis	Which is worse rheumatoid arthritis or osteoarthritis
How rheumatoid arthritis starts	When rheumatoid arthritis is fatal	Are rheumatoid arthritis and polymyalgia rheumatica related	Will rheumatoid arthritis go away	Why rheumatoid arthritis causes anaemia	What rheumatoid arthritis symptoms	Can rheumatoid arthritis affect your neck	Where does rheumatoid arthritis affect the body	Who does rheumatoid arthritis affect	
How rheumatoid arthritis is diagnosed	When does rheumatoid arthritis start	Are rheumatoid arthritis patients immunocompromised	Will rheumatoid arthritis kill me	Why rheumatoid arthritis is called autoimmune disease	What rheumatoid arthritis looks like	Can rheumatoid arthritis be cured	Where is rheumatoid arthritis	Who treat rheumatoid arthritis	
How rheumatoid arthritis affects movement	When is rheumatoid arthritis diagnosed	Are rheumatoid arthritis patients candidates for dental implants	Will rheumatoid arthritis cripple me	Why rheumatoid arthritis common in females	What rheumatoid arthritis drugs are covered by medicare	Can rheumatoid arthritis kill	Where is rheumatoid arthritis pain		
How rheumatoid arthritis can be cured	When is rheumatoid arthritis	Are rheumatoid arthritis and ankylosing spondylitis related	Will rheumatoid arthritis spread	Why rheumatoid arthritis causes fatigue	What’s rheumatoid arthritis	Can rheumatoid arthritis cause anaemia	Where is rheumatoid arthritis common		
How rheumatoid arthritis affects the heart	When is rheumatoid arthritis considered a disability	Are rheumatoid arthritis and lupus related	Will rheumatoid arthritis cause hair loss	Why does rheumatoid arthritis cause fatigue	What rheumatoid arthritis risk factors	Can rheumatoid arthritis cause weight loss	Where do you get rheumatoid arthritis		
How rheumatoid arthritis is caused	When is rheumatoid arthritis caused	Are rheumatoid arthritis and rheumatic fever related	Will rheumatoid arthritis cause weight loss	Why does rheumatoid arthritis make you tired	What rheumatoid arthritis symptoms	Can rheumatoid arthritis be inherited	Where to live with rheumatoid arthritis

**Table 3:** Top: the most common search terms on “rheumatoid arthritis,” using the prepositions: with, without, can, to, is, for, near, bottom: the eight most common search terms on “rheumatoid arthritis,” using the comparators: vs, and, like, or, versus

Searches on “rheumatoid arthritis”: prepositions						
With	Without	Can	To	Is	For	Near
Rheumatoid arthritis with coronavirus	Rheumatoid arthritis without swelling	Rheumatoid arthritis can it kill you	Rheumatoid arthritis to lupus	Rheumatoid arthritis is it hereditary	Rheumatoid arthritis for dummies	Rheumatoid arthritis near me
Rheumatoid arthritis with fibromyalgia	Rheumatoid arthritis without rheumatoid factor	Rheumatoid arthritis can it be cured	Rheumatoid arthritis to hands	Rheumatoid arthritis is an autoimmune disease	Rheumatoid arthritis for child	Rheumatoid arthritis doctors near me
Rheumatoid arthritis with COVID-19	Rheumatoid arthritis without medication	Rheumatoid arthritis can you die	Rheumatoid arthritis to lungs	Rheumatoid arthritis is it dangerous	Rheumatoid arthritis for dogs	Rheumatoid arthritis doctor near me
Rheumatoid arthritis with negative rheumatoid factor	Rheumatoid arthritis without joint pain	Rheumatoid arthritis can it go away	Rheumatoid arthritis to ankle	Rheumatoid arthritis is it inherited	Rheumatoid arthritis for nurses	Rheumatoid arthritis specialist near me
Rheumatoid arthritis with psoriasis	Rheumatoid arthritis without inflammation	Rheumatoid arthritis can it affect the lungs	Rheumatoid arthritis to Chinese	Rheumatoid arthritis is it serious	Rheumatoid arthritis for young adults	Rheumatoid arthritis support group near me
Rheumatoid arthritis with normal rheumatoid factor	Rheumatoid arthritis without pain	Rheumatoid arthritis can I still work	Rheumatoid arthritis to neck	Rheumatoid arthritis is there a cure	Rheumatoid arthritis for medical students	Best doctor for rheumatoid arthritis near me
Rheumatoid arthritis with normal ESR and CRP	Rheumatoid arthritis without fever	Rheumatoid arthritis can it affect eyes	Rheumatoid arthritis to	Rheumatoid arthritis is it genetic	Rheumatoid arthritis for doctors	
Rheumatoid arthritis with Crohn’s disease	Rheumatoid arthritis without inflammatory markers	Rheumatoid arthritis can you get disability	Rheumatoid arthritis in Hindi	Rheumatoid arthritis is this a disability	Rheumatoid arthritis for exercises	

Searches on “rheumatoid arthritis”: comparators				
Vs	And	Like	Or	Versus
Rheumatoid arthritis vs gout	Rheumatoid arthritis and COVID	Rheumatoid arthritis like diseases	Rheumatoid arthritis or lupus	Rheumatoid arthritis versus arthritis
Rheumatoid arthritis vs lupus	Rheumatoid arthritis and osteoarthritis	Rheumatoid arthritis like syndrome	Rheumatoid arthritis or gout	Rheumatoid arthritis versus osteoarthritis
Rheumatoid arthritis vs osteoarthritis	Rheumatoid arthritis and shielding	Rheumatoid arthritis like symptoms	Rheumatoid arthritis or MS	Rheumatoid arthritis versus fibromyalgia
Rheumatoid arthritis vs osteoarthritis X-ray	Rheumatoid arthritis and eyes	Rheumatoid arthritis like polyarthritis	Rheumatoid arthritis or osteoarthritis	Rheumatoid arthritis versus lupus
Rheumatoid arthritis vs other arthritis	Rheumatoid arthritis and alcohol	Rheumatoid like arthritis	Rheumatoid arthritis or ankylosing spondylitis	Rheumatoid arthritis versus gout
Rheumatoid arthritis vs osteoarthritis hands	Rheumatoid arthritis and exercise	Rheumatoid arthritis feels like	Rheumatoid arthritis or arthritis	Rheumatoid arthritis versus psoriatic arthritis
Rheumatoid arthritis vs reactive arthritis	Rheumatoid arthritis and pip	Rheumatoid arthritis flu like symptoms	Rheumatoid arthritis or psoriatic arthritis	Rheumatoid arthritis versus MS
Rheumatoid arthritis vs psoriatic arthritis	Rheumatoid arthritis and fatigue	Rheumatoid arthritis feels like flu	Rheumatoid arthritis or something else	Rheumatoid arthritis versus osteoarthritis symptoms

Searches are ordered in popularity vertically down each column, with the most popular at the top

Tables 2 and 3 display the results for search terms relating to questions, prepositions, and comparisons to the term “rheumatoid arthritis,” in the same manner as for “arthritis.”

Public searches performed on these topics predominantly relate to impacts on quality of life, relief of debilitating features including pain, and prognosis. On review of questions asked on arthritis, approximately 30% referred to debilitating features or quality of life, while just over 20% were on treatment or prognosis. In the prepositions’ category, approximately 20% of searches were on debilitating features or quality of life, with a further 20% on treatment or prognosis.

For on “rheumatoid arthritis” searches, approximately 20% of search questions were on debilitating features or quality of life, with approximately 30% on treatment or prognosis. Looking at searches on rheumatoid arthritis, by prepositions, approximately 15% were on debilitating features or quality of life, with 30% on treatment or prognosis. The remainder of searches for both arthritis and rheumatoid arthritis covered topics including but not limited to anatomical sites of symptoms, possible associations with other rheumatological diseases (e.g. gout, systemic lupus erythematosus), investigations and diagnosis, and symptoms in animals.

## Discussion

We used an openly available online tool to investigate Google searches for the terms “arthritis” and “rheumatoid arthritis,” conducted by the public. Our results reveal striking patterns in search themes, with a focus on treatment, prognosis, quality of life, and debilitating features, including impact on work. While some results were novel, consistencies were seen with previous research in these areas, such as quality of life and patient-reported outcomes. These similarities with traditional methods of PPI suggest a role for a search query tool in this area.

One of the most common themes in searches for both arthritis and rheumatoid arthritis was on treatment. Specifically, some of the most frequently searched questions were “can arthritis be cured?”, “can arthritis be treated?”, “will arthritis go away?”, and “rheumatoid arthritis- can it be cured?”, as well as variations on these questions. This is consistent with previous studies on patient-reported health service needs. A recent scoping review identified that patients with inflammatory arthritis value discussions on treatment outside conventional medicine, such as alternative and complimentary therapies and dietary advice, as well as practical tools to help with daily activities [8]. Indeed, our review of Google searches revealed the following as some of the commonest searches: “arthritis and diet” and “arthritis can opener”, consistent with the findings in this review and elsewhere in the literature [9, 10].

Another common theme in the searches centred on quality of life, and debilitating aspects of arthritis relating to this. Some specific frequently-searched examples include “when arthritis becomes debilitating”, “will rheumatoid arthritis cripple me?”, and “what rheumatoid arthritis feels like”. Quality of life outcomes have been explored increasingly in recent years in the rheumatic diseases. This has sought to increase clinical awareness of the burden of disease on mental health and daily living, with recommendations for factors such as health-related quality of life and fatigue to be routinely assessed in those with conditions including rheumatoid arthritis [11–13]. Our review of searches reveal that this remains an important priority in the public domain.

In addition, some specific frequent searches relating to quality of life, revealed public interest in aspects relating to work and arthritis. These included such examples as: “arthritis- can I claim PIP?”, “rheumatoid arthritis- can you work?”, and “is rheumatoid arthritis a disability?”. We know from previous studies and patient-reported outcomes that productivity at work, as well as presenteeism and absenteeism, are affected by all forms of arthritis [14–16]. However, these searches demonstrate more sensitive topics, such as eligibility for benefits, that patients may not feel able or comfortable to discuss during appointments. This highlights the need for clinicians to facilitate conversations regarding work and disability during clinic appointments, and the importance of the multi-disciplinary team, including psychologists and occupational therapists, and patient support services.

Patient and public involvement, in rheumatology and elsewhere, has repeatedly highlighted the value placed on their relationship with health professionals, including ease of communication and approachability [8, 17]. Consistent with this, some common Google searches included “rheumatoid arthritis doctors near me” and “rheumatoid arthritis support group near me”. This again demonstrates the importance of access to patient, as well as clinician, support.

The co-existence of fibromyalgia with other rheumatic diseases has become more apparent in recent years, associated with poorer clinical outcomes in inflammatory arthritis [18, 19]. Some of the most common public searches seek to explore this relationship e.g. “arthritis versus fibromyalgia” and “are arthritis and fibromyalgia connected?”. These searches may also represent questions asked by patients during the diagnostic process for inflammatory arthritis or fibromyalgia, as they seek to explore the overlapping symptoms and relationship between the two diseases. This is also consistent with studies demonstrating a sense of ‘invalidation’ experienced by those with fibromyalgia, due to the invisibility and medically-unexplained nature of the syndrome, and the subsequent impact on quality of life outcomes in these patients [20, 21].

Some searches did not follow a theme, or revealed unexpected patterns of search behaviour. One of the commonest phrases was “rheumatoid arthritis and COVID”, consistent with the time at which we conducted this study (i.e. during the UK lockdown and shielding period for those at high-risk of complications of COVID-19, including some patients with rheumatoid arthritis). Other commons searches were on the genetics of rheumatoid arthritis (“is rheumatoid arthritis hereditary?” and “rheumatoid arthritis-is it genetic?”). This perhaps demonstrates a sensitive area which clinicians may not routinely address in clinic, but of importance to the patient. On a similar theme, searches such as “arthritis and alcohol” and “arthritis and sex” reveal similar sensitive topics which may prove potentially embarrassing for the patient to ask, leading them to seek answers on the Internet and online forums.

### Future directions

Our use of a search query tool to explore searches conducted on “arthritis” and “rheumatoid arthritis” reveal known concerns from patients and the public, but also demonstrate areas of concern which may not be immediately apparent to researchers and clinicians. There is, therefore, scope to apply this tool as an adjunct to traditional methods of PPI, for example, when planning research in sensitive fields, such as opioid addiction and medication side-effects, for example, corticosteroids. It may also enhance and encourage research in areas including the impact of rheumatic disease on mental health. In the era of COVID-19 and telemedicine, such a tool provides a quick method of ascertaining public concerns and questions on its use, not only in rheumatology, but also other fields. Finally, as our internet and social media habits change, search query tools which are able to capture phrases used on social media may provide additional information on patient perceptions on healthcare-related topics. This has already been done through applications which are able to capture increased social media activity in relation to disease activity, including pain, but there is scope to extend further to incorporate use of specific phrases [22].

### Limitations

A key limitation of such a tool is the inability to attribute all searches to a patient body. However, the tool can provide broad indications of public perceptions of a topic, within a given geographical region, e.g. UK. Furthermore, while searches on a topic such as arthritis may not change significantly from one day to another, searches conducted in Google on current and fast-changing situations may do so, which will affect the output from search query tools if conducted at two separate times. A prime example of this is search terms relating to COVID-19. In addition, as described above, search query tools are currently limited in number and can only capture data from the most commonly-used search engine, Google. Search query tools are complimentary, but not identical, to online platforms such as Google Trends which is able to display volumes and patterns of searches on a given topic, but no granularity on the nature of these searches. It is this additional information on extended phrases and questions entered into search engines which may enable their use as an adjunct to traditional PPI. As their utility grows in areas outside marketing, including in healthcare, possibly so too will their scope, including in areas such as social media.

## Conclusion

PPI is, and must continue to be, an essential part of research, from inception, to implementation, and dissemination of results. However, we have demonstrated the utility of a search query tool to complement traditional methods of PPI, for example, to conduct scoping searches at a project’s inception, or in addition to PPI, particularly at present, when focus groups may be difficult to convene due to social distancing regulations. Not only are the searches revealed by this tool consistent with known priorities of patients and the public, but they also demonstrate areas which benefit from user anonymity, such as concerns regarding the impact of arthritis on relationships, lifestyle, and work. This has the potential to guide future research questions, increasing the alignment of research priorities with those of the public.
